# Mosaic fungal individuals have the potential to evolve within a single generation

**DOI:** 10.1038/s41598-020-74679-5

**Published:** 2020-10-19

**Authors:** Maura G. Tyrrell, Diane C. Peabody, Robert B. Peabody, Magdalena James-Pederson, Rachel G. Hirst, Elisha Allan-Perkins, Heather Bickford, Amy Shafrir, Robert J. Doiron, Amber C. Churchill, Juan Carlos Ramirez-Tapia, Benjamin Seidel, Lynes Torres, Kathryn Fallavollita, Thomas Hernon, Lindsay Wiswell, Sarah Wilson, Erica Mondo, Kathleen Salisbury, Carrie Peabody, Patrick Cabral, Lauren Presti, Kelsey McKenna-Hoffman, Michele Flannery, Kaitlin Daly, Darius Haghighat, Daniel Lukason

**Affiliations:** Biology Department, Stonehill College, Easton, MA 02357 USA

**Keywords:** Evolution, Ecology, Evolutionary ecology, Genetics, Evolutionary biology, Haplotypes, Heritable quantitative trait

## Abstract

Although cells of mushroom-producing fungi typically contain paired haploid nuclei (n + n), most *Armillaria gallica* vegetative cells are uninucleate. As vegetative nuclei are produced by fusions of paired haploid nuclei, they are thought to be diploid (2n). Here we report finding haploid vegetative nuclei in *A. gallica* at multiple sites in southeastern Massachusetts, USA. Sequencing multiple clones of a single-copy gene isolated from single hyphal filaments revealed nuclear heterogeneity both among and within hyphae. Cytoplasmic bridges connected hyphae in field-collected and cultured samples, and we propose nuclear migration through bridges maintains this nuclear heterogeneity. Growth studies demonstrate among- and within-hypha phenotypic variation for growth in response to gallic acid, a plant-produced antifungal compound. The existence of both genetic and phenotypic variation within vegetative hyphae suggests that fungal individuals have the potential to evolve within a single generation in response to environmental variation over time and space.

## Introduction

Evolution in unitary organisms such as fish, frogs, and most other animals occurs over generations; and its rate is related inversely to generation times. In modular organisms such as sponges, corals, and many plants and fungi, individual genets might have very long generation times; is evolution consequently a much slower process? Here we show that in *A. gallica*, long-lived mosaic fungal genets have the potential to evolve within a single generation.

Vegetative cells of many fungi contain single haploid (n) nuclei. However, those of higher fungi typically contain pairs of haploid nuclei, so their cells are described as dikaryotic (n + n). Because allelic expression can be masked or altered in diploid (2n) nuclei, it has been proposed that haploid and dikaryotic fungi have selective advantages over diploid plants or animals^1–3^.

Among higher fungi that produce mushrooms (basidiomycetes), the dikaryotic (n + n) stage ends when haploid nuclei fuse to form single diploid nuclei (2n) in basidial cells on mushroom gills. Basidial meiosis then produces 4 haploid spores (n) that germinate to produce primary mycelia (n) capable of mating with other compatible primary mycelia (n) to reestablish dikaryons (n + n). However, *A. gallica*’s life cycle is unusual among basidiomycetes, because after spore formation a second diploidization-haploidization event occurs. After compatible primary mycelia (n) fuse to reestablish dikaryons (n + n), nuclear fusion produces diploid nuclei (2n) that undergo a second (vegetative-stage) haploidization at some point prior to mushroom formation^4–8^. The resulting haploid nuclei (n) persist in vegetative stages (soil mycelia, rhizomorphs) and in mushroom stipes^7^.

It has been proposed that the vegetative-stage diploidization-haploidization in *A. gallica*’s life cycle produces haploid genetic mosaics^4–8^, and that, if haploid genetic mosaicism (HGM) is found in other *Armillaria* species, it might account for some of the broad range of ecological, morphological, and pathogenic diversity that characterizes the genus. *Armillaria* species occur in boreal, temperate, and tropical forests where they can act as beneficial soil-borne decomposers, mycorrhizal symbionts with trees and several orchid species, or economically important root rot pathogens infecting over 600 plant species^9^. Some individuals (or genets) of *A. gallica* are among the world’s largest and oldest living organisms, with one individual in Michigan, USA, estimated to cover 75 hectares, weigh about 4 × 10^5^ kg, and have lived for 2500 years^10,11^. The hyphae of *A. gallica*’s diffuse filamentous soil mycelia and dark strap-like rhizomorphs are important in nutrient acquisition and vegetative spread^12^, while short-lived, above-ground mushrooms accomplish long-range spore dispersal in air currents^13^.

Our earlier studies^7,8^ are consistent with the existence of HGM in *A. gallica*; but analyses were limited to isozyme loci, mating-type loci, and a restriction fragment length polymorphism (RFLP) of *IGS-1* in stipes. *IGS-1* analyses were instructive but limited to one locus in one tissue type of a single individual^7,8^. In the present study RFLP analyses were expanded to include five loci in three tissue types from seven geographically distinct sites in southeastern Massachusetts, USA. We used somatic incompatibility testing^14^ to establish that collections from all 7 sites represent distinct genets. Because *EF1α* exists as a single-copy gene in *Armillaria*^15,16^, multiple cloned, single-copy *EF1α* sequences were isolated from individual hyphal filaments to confirm HGM within and among hyphae that form rhizomorphs. Cytoplasmic bridges connecting hyphae that could provide a mechanism for maintaining HGM were observed in field-collected and cultured rhizomorph samples. Although haploidy had been established previously in spore and vegetative stages of these genets, microspectrophotometry was used to confirm haploidy in the cultured spore and rhizomorph samples used in the current growth studies. Therefore, in addition to the haploid condition that may give other fungi advantages over diploid organisms, HGM may provide *A. gallica* with a source of genetic variation not yet documented in vegetative stages of other fungi. Growth studies of *A. gallica* provide evidence of phenotypic variation among and within rhizomorph hyphae grown in eight concentrations of the antifungal compound, gallic acid. Taken together, haploid genetic mosaic variation, phenotypic variation for a fitness-related trait, and the presence of cytoplasmic bridges that could maintain HGM suggest that *A. gallica* has the potential to undergo adaptive change within the mycelium of a single individual within a single generation.

## Results

### Haploidy of rhizomorph hyphal filament lines collected in Raynham and Bridgewater, MA

Raynham spore nuclei (N = 100), soil mycelium nuclei (N = 100), and rhizomorph hyphal filament nuclei (N = 100) were shown to be haploid in an earlier study of *A. gallica* growth responses to water potential^8^. To confirm haploidy of the current study’s cultured rhizomorph hyphal filament lines, multiple nuclei were measured for nuclear DNA content in each of 10 lines in the Raynham and Bridgewater genets. Microspectrophotometric measurement permitted in situ observation of DAPI-stained nuclei with phase contrast microscopy before measurements and with fluorescence microscopy after measurements. This made it possible to confirm life cycle stages of nuclei prior to measurement and to verify that no other nuclei had been close enough to contribute fluorescence to the measurement. Pre- and post-measurement observation also made it possible to confirm the uninucleate condition of each cell.

**Figure 1:**
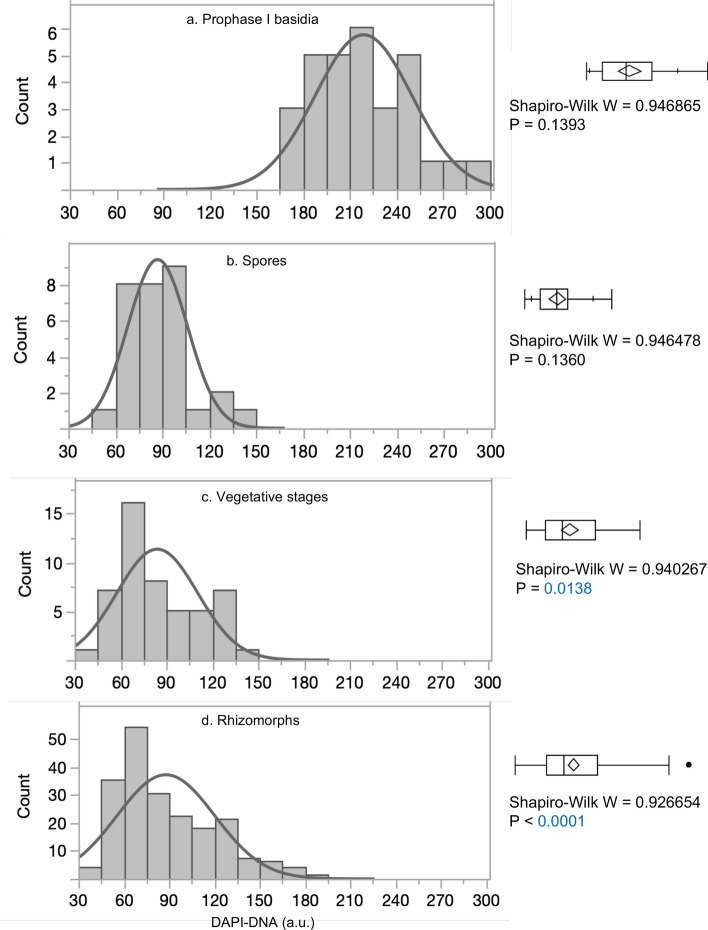
DAPI-DNA values for *A. gallica* nuclei show spores and vegetative nuclei are haploid, in contrast to the diploid nuclei from prophase I basidia. (**a**). Field-collected prophase I basidia (N = 30). (**b**). Field-collected spores (N = 30). (**c**). Field-collected vegetative stages (rhizomorphs and soil mycelia, N = 50). (**d**). Cultured rhizomorphs (N = 202). Shapiro–Wilk W goodness-of-fit test: H_o_ = normal distribution; small P-values reject H_o_.

A total of 202 cultured rhizomorph nuclei were measured (11 nuclei were measured for each of 2 lines; 10 nuclei were measured for each of 18 lines). For comparison, cultured lines were compared to samples fixed in 95% ethanol within 2 h of collection from nature. These samples included 30 prophase I basidia nuclei, 30 spore nuclei, and 50 rhizomorph and soil mycelia (vegetative-stage) nuclei^8,17,18^ (Supplementary Table S1). Because vegetative-stage nuclei and cultured rhizomorph nuclei were not normally distributed (Fig. 1c,d), even when log- or square-root transformed, neither standard nor Welsh’s ANOVA could be used to compare means of the four groups; because variances were not similar, the means could not be compared by the Kruskal–Wallis test. However, prophase I basidia nuclei and spore nuclei were normally distributed (Fig. 1a,b); and a t-test showed that prophase I basidia nuclei had significantly more DNA than spore nuclei (*P* < 0.0001). This established expected distributions and DNA content for diploid (prophase I basidia) and haploid (spore) nuclei. Figure 1 and Supplementary Table S1 show that total DNA content of vegetative-stage nuclei (Fig. 1c, mean ± sd = 84 ± 31) and cultured rhizomorph nuclei (Fig. 1d, mean ± sd = 83 ± 22) are close to the total DNA content of spore nuclei (Fig. 1b, mean ± sd = 87 ± 22), with the primary difference being that vegetative-stage nuclei and cultured rhizomorph nuclei have distributions skewed to the right. This is expected given that vegetative stages, whether in nature or in the lab, are expected to be growing and to include both unreplicated (1C) and replicated (2C) haploid nuclei. Figure 1 shows that all three samples of putative haploid-stage nuclei (Fig. 1b–d) contain less DNA than prophase I basidial nuclei (Fig. 1a, mean ± sd = 219 ± 31) To verify that most cultured-rhizomorph-line hyphal compartments are uninucleate and establish that they might be expected to contain both replicated and unreplicated nuclei, we examined a set of 197 cultured rhizomorph hyphal filament compartments: 186 were uninucleate, 9 contained division figures, and 2 were binucleate. Cultured rhizomorph nuclei are therefore considered to be haploid with a large peak near the 1C area and a smaller number of additional values in the 2C range (Fig. 1d).

### RFLP patterns for individuals collected in multiple towns

**Figure 2:**
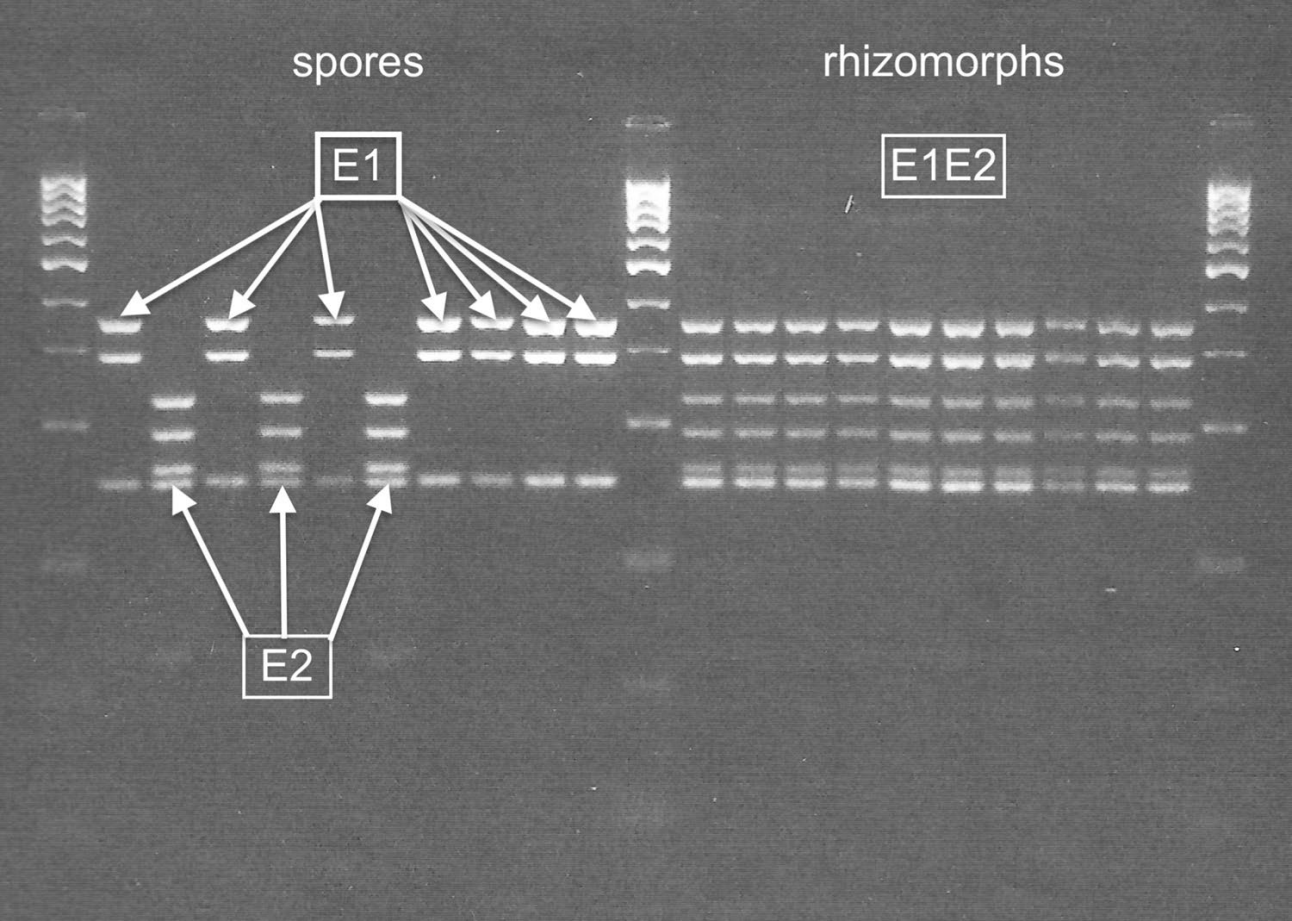
*EF1α* x *HaeIII* RFLP patterns for Raynham differ for spores and rhizomorphs. Spores have either allele E1 (lanes 2, 4, 6, 8–11) or allele E2 (lanes 3, 5, 7). Rhizomorphs have both alleles (pattern E1E2, lanes 13–22). Lanes 1, 12, and 23 are markers.

Our conclusion of haploidy for vegetative stages and spores used in growth experiments is based on DAPI-DNA measurements. Because this conclusion is at odds with commonly held expectations of diploidy for vegetative stages in *Armillaria*, we conducted a Restriction Fragment Length Polymorphism (RFLP) survey of 7 southeastern Massachusetts genets (Supplementary Table S2). The most common RFLP patterns for polymorphic spore, stipe, or rhizomorph lines are represented in Fig. 2. Polymorphic spores always have one or the other of two alleles, whereas polymorphic stipes and rhizomorphs most frequently have a pattern that combines both alleles. These patterns are consistent with either of two alternative interpretations: (1) spores are haploid; stipes and rhizomorphs are heterozygous diploids, or (2) spores are haploid; stipes and rhizomorphs are haploid genetic mosaics.

### EF1α sequences

Baumgartner^15^ used Southern blotting to establish *EF1α* as a single-copy gene in *Armillaria mellea.* Sipos et al. subsequently used PacBio and Illumina sequencing to show that *EF1α* is a single-copy gene in *A. gallica*^16^. Because RFLP patterns did not allow us to determine whether rhizomorphs are heterozygous diploids (having two haplotypes) or haploid genetic mosaics (having more than two haplotypes), we decided to distinguish between these two models by cloning and sequencing multiple copies of *EF1α* genes isolated from single hyphal filaments. Table 1 and Supplementary Table S3 list *EF1α* sequences for 45 rhizomorph hyphal filament lines isolated from genets in Raynham, Norton, N. Easton, and Milton. In 19 of 45 lines only 1 or 2 clones were successfully sequenced, so their sequences could not distinguish between models. In 26 of 45 lines, however, 3 or more clones were successfully sequenced. In 11 of these 26 lines, clones had only 1 or 2 haplotypes; but in 15 of the 26 lines, clones had either 3 or 4 haplotypes, and therefore made it possible to distinguish between models. Raynham had 5 lines in which multiple clones had 3 haplotypes, and 2 lines in which multiple clones had 4 haplotypes. Norton had 5 lines in which multiple clones had 3 haplotypes, and 1 line in which multiple clones had 4 haplotypes. North Easton and Milton each had 1 line in which multiple clones had 3 haplotypes. Because these 15 rhizomorph hyphal filament lines had either 3 or 4 different haplotypes for a single-copy gene, they could not have been heterozygous diploids. This finding allows us to reject the heterozygous diploid model and accept the haploid genetic mosaic model, if only for these 4 genets.

**Table 1:**
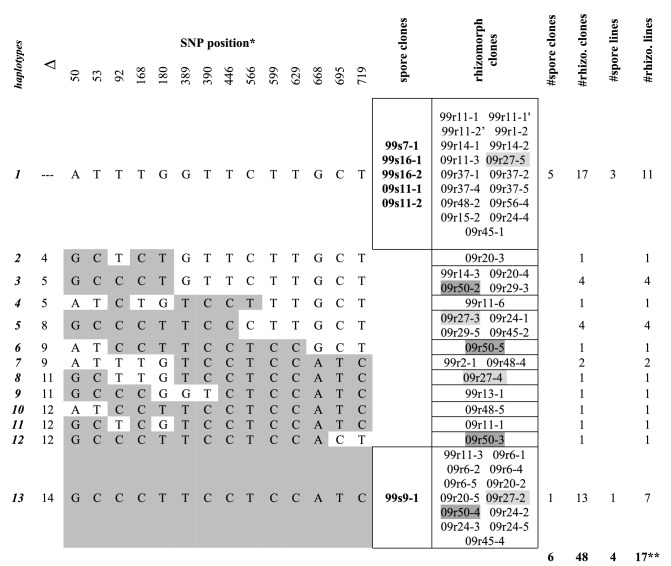
*EF1α* haplotypes of rhizomorphs are more variable than those of spores in the Raynham genet.

All SNP’s differing from those of haplotype 1 are shaded and the count is shown in column 2 (∆). For each clone, the notation sequence is year of collection (1999 or 2009), then source (s for spore or r for rhizomorph), then cell or hyphal filament line number, then clone number after the hyphen. Similar data for Norton, N. Easton, and Milton genets are in Supplementary Table S3.

*Numbering is from the start of the amplified region.

**Some rhizomorph hyphal lines have multiple SNP haplotypes: 99r14, 09r11, and 09r29 have two; 99r11, 09r20, 09r24, 09r45, and 09r48 have three; 09r27 (pale gray) and 09r50 (dark gray) have four. No spore cell lines have multiple SNP haplotypes.

Table 1 and Supplementary Table S3 list *EF1α* sequences for 25 spore cell lines isolated from the same 4 genets. We sampled fewer spore cell lines and clones of spore cell lines than we did for rhizomorph hyphal filament lines because spores are widely accepted as being haploid. Nine spores had 1 clone, 8 spores had 2 clones, 7 spores had 3 clones, and 1 spore had 4 clones. Of the 16 spores where 2 or more clones were sequenced, only 2 had more than one haplotype (Raynham had 0; Norton had 0; N. Easton had 1 spore [s16] with 2 haplotypes that differed from one another for $\mathrm{only}$ 1 of 48 SNPs; Milton had 1 spore [s6] with 2 haplotypes that differed at 22 of 23 SNPs).

Each row in Table 1 (Raynham genet) and Supplementary Table S3 (Norton, N. Easton, and Milton genets) represents an *EF1α* haplotype that was sequenced in 1 of these 4 genets. Raynham had 13 haplotypes; Norton had 11 haplotypes; N. Easton had 12 haplotypes; Milton had 12 haplotypes. For each genet, haplotypes that differ most from one another are positioned in the top and the bottom rows of their respective tables. Haplotypes more closely resembling haplotype 1 are listed closer to the top of the table; haplotypes differing more from haplotype 1 are listed closer to the bottom of the table. Row position therefore reflects total number of single nucleotide polymorphism (SNP) differences relative to haplotype 1. This arrangement reveals clustering patterns that distinguish spores from rhizomorphs. Combining spore data for all 4 genets, 98% of all *EF1α* haplotypes (50 of 51 clones) are located in either the top 2 or bottom 2 rows of their respective tables; and only 2% (1 of 51 clones) are located in intermediate rows. In contrast, for rhizomorphs from all 4 genets, only 45% of *EF1α* haplotypes (63 of 139 clones) are located in the top 2 or bottom 2 rows of their respective tables; while 55% (76 of 139 clones) are located in intermediate rows. Interestingly, in 3 of 4 genets, spore and rhizomorph clones do not share any haplotypes.

### Cytoplasmic bridges

**Figure 3:**
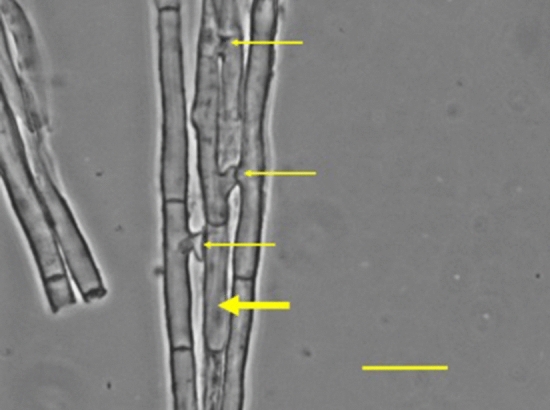
Cytoplasmic bridges (thin arrows) connect one hypha (thick arrow) to 3 nearby hyphae in a rhizomorph fixed in 95% ethanol upon collection from the field. Bar = 10 μM.

**Figure 4:**
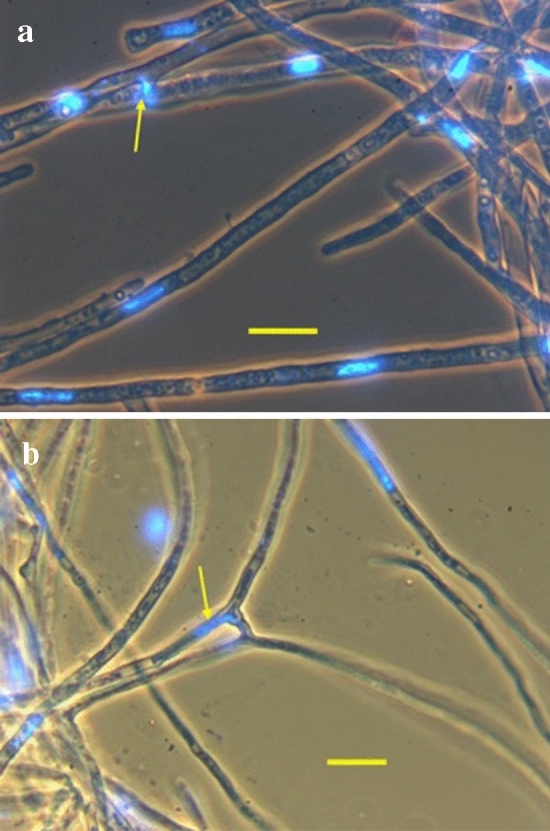
DAPI staining shows nuclei (arrows) in cytoplasmic bridges between hyphae grown from rhizomorph hyphal filament tip isolates. (**a**) Nucleus within a bridge. (**b**) Nucleus entering or exiting a bridge. Bars = 10 μM.

Rhizomorph samples fixed in 95% ethanol within 2 h of collection from nature had cytoplasmic bridges that frequently connected multiple hyphae (Fig. 3). Rhizomorph hyphal filament lines grown in culture were typically monokaryotic; cytoplasmic bridges averaging ~ 10 µM in length were common, and nuclei were frequently seen in or near them (Fig. 4). Spore cell lines lacked cytoplasmic bridges.

### Growth studies

**Figure 5:**
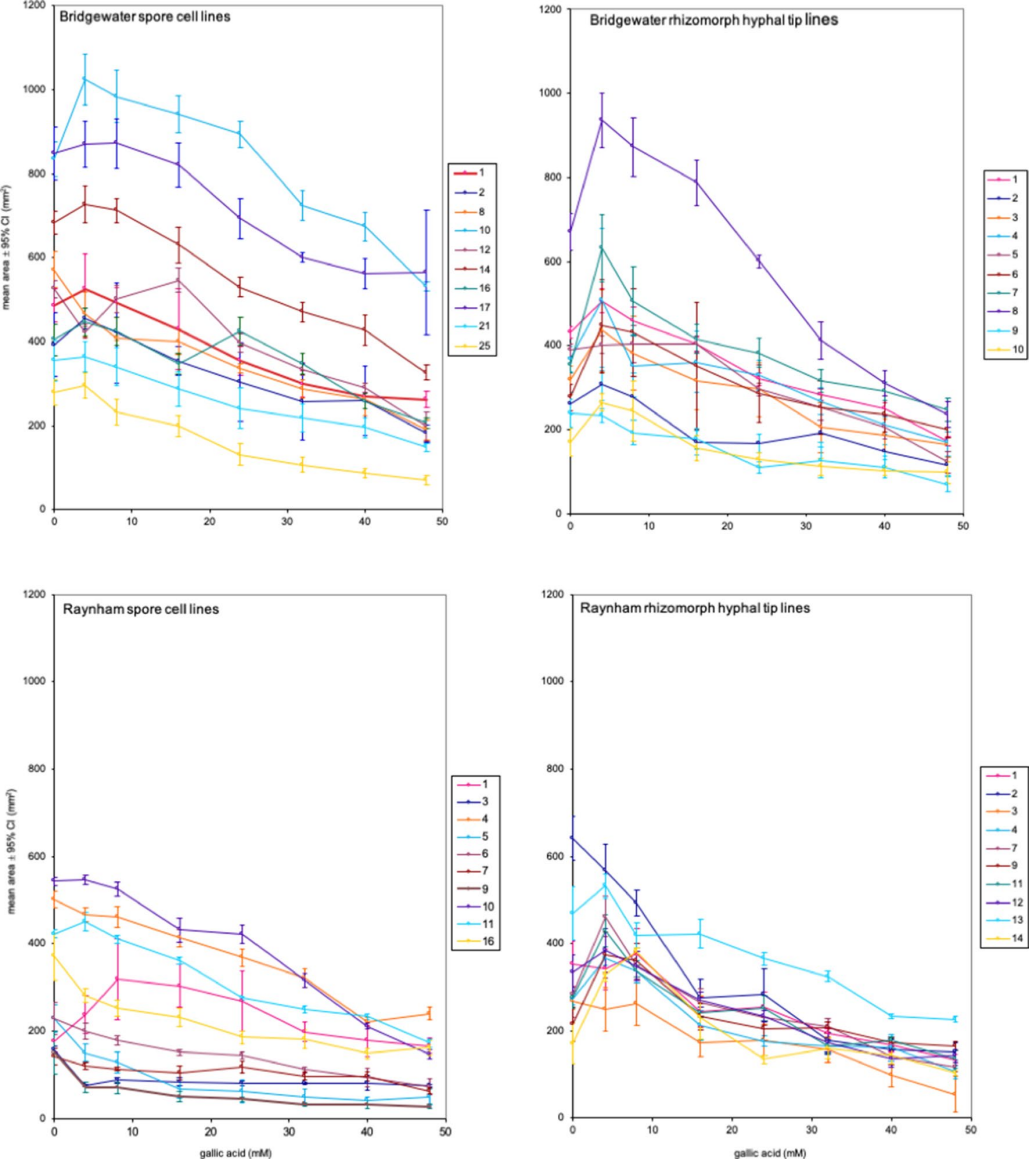
Reaction norm lines show that spore cell lines and rhizomorph hyphal filament lines from single genetic individuals from Bridgewater and Raynham, MA, differed for both growth and phenotypic plasticity. All ANOVA *P*-values were significant (P < 0.0001) for line effects (growth differences among lines), treatment effects (effect of gallic acid concentration on growth), and line × treatment effects (phenotypic plasticity for spore and rhizomorph lines). F-values and degrees of freedom are listed in Supplementary Table S6. N = 1571 culture plates, 1547 with independent environmental histories (Supplementary methods).

Bark-extract vs. wood-extract growth experiments were conducted to explore the possibility that HGM might affect growth and phenotypic plasticity. Differences were significant for 8 of 8 cell line effects, 6 of 8 treatment effects, and 7 of 8 cell line $\times$ treatment effects (Supplementary Table S4, Supplementary Fig. S1). These effects suggest that HGM has the potential to affect growth and phenotypic plasticity in nature. However, since crude bark extracts and wood extracts are complex mixtures, it was not possible to determine which component(s) induced the observed responses (Supplementary methods). We therefore shifted our focus to growth effects of defined media containing known concentrations of plant-produced, defensive phenolic compounds. Preliminary tests showed cell-line growth was affected by exposure to catechin, ellagic acid, gallic acid, and vanillin. Based on results, we selected gallic acid as the treatment for a growth experiment comparing spore cell lines and rhizomorph hyphal filament lines from Bridgewater and Raynham, MA. Variance in growth was greater for spore cell lines than for rhizomorph hyphal filament lines in both the Bridgewater (*P* < 0.0001) and Raynham (*P* = 0.0037) genets (Supplementary Table S5); and all cell line effects, treatment effects, and cell line $\times$ treatment effects were significant for both cell types in both genets (*P* < 0.0001, Fig. 5, Supplementary Table S6). Significant treatment effects indicate that gallic acid concentration affected growth. Significant cell line effects indicate that different cell lines grew differently in response to gallic acid. Significant cell line $\times$ treatment effects indicate that cell lines differed from one another in phenotypic plasticity. Differences in reaction norm lines in Fig. 5 visually represent the differences in growth and phenotypic plasticity that exist among cell lines. These quantitative-trait differences among the cell lines of single individuals suggest that selection has the potential to affect growth and phenotypic plasticity in nature.

## Discussion

### Within-generation HGM

After matings of compatible hyphal tips grown from spores, haploid dikaryotic nuclei (n + n) of *A. gallica* fuse to produce diploid monokaryons (2n). As monokaryons are persistent in vegetative stages and often possess two distinct molecular-marker alleles, the model of vegetative heterozygous diploidy is widely accepted. But since other studies show vegetative stages can possess recombinant, haploid nuclei, an alternative hypothesis has been advanced. This hypothesis proposes a life cycle in which a vegetative-stage haploidization produces HGM^6,7,17,18^. Our analyses confirm that vegetative-stage hyphae can be haploid (Fig. 1, Supplementary Table S1), while still possessing two different molecular-marker alleles (Supplementary Table S2).

**Figure 6:**
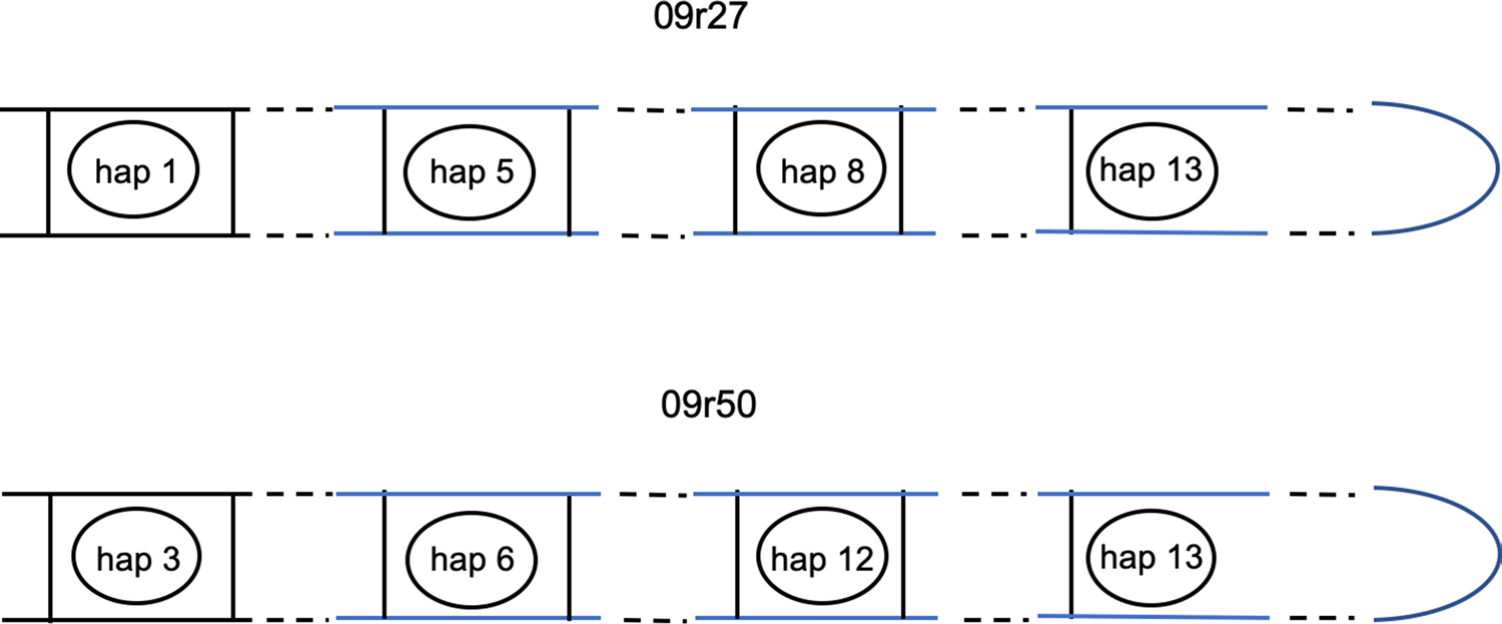
Haploid Genetic Mosaicism is exemplified in two rhizomorph hyphal filament lines (09r27 and 09r50) isolated from the Raynham genet. The mycelium containing hyphae with these haplotypes exhibits both within-line and among-line nuclear heterogeneity.

Although RFLP data are consistent with both heterozygous diploid and haploid genetic mosaic models, DNA content data and *EF1α* sequence data both argue against the heterozygous diploid model. Since *EF1α* is a single-copy gene, multiple cloned sequences isolated from a single hyphal filament should have only 1 haplotype if the filament is a diploid homozygote or 2 haplotypes if it is a diploid heterozygote; but it could have 1, 2, 3 or more haplotypes if it is a haploid genetic mosaic. The upper limit on the number of haplotypes detected in a hyphal filament is set by the number of hyphal compartments recovered during cell-line isolation. We estimate that, on average, six contiguous compartments were harvested each time we isolated a hyphal filament line; and there were 26 instances in which 3 or more clones were successfully sequenced from within a single hyphal filament line. In these 26 lines, we detected 1 or 2 haplotypes 11 times and 3 or 4 haplotypes 15 times (Table 1, Supplementary Table S3a–c). The 11 instances in which 1 or 2 haplotypes were detected are compatible with either model; but the 15 instances in which 3 or 4 haplotypes were detected are compatible with only the haploid genetic mosaic model. In conjunction with the finding of haploidy in vegetative stages, this finding argues against the heterozygous diploid model and supports the haploid genetic mosaic model. We define a haploid genetic mosaic as a mycelium with haplotypes that vary within and among hyphae. As an example, Fig. 6 depicts two haploid genetic mosaic rhizomorph hyphal filament lines that were isolated from the Raynham genet.

Haplotype designations **hap 1, hap 3…hap 13** refer to *EF1α* haplotypes listed in rows 1, 3, 5, 6, 8, 12, and 13 of Table 1. Note that (1) haplotype 13 is the only haplotype shared by both filament lines; (2) the order of the nuclei in the filaments is not known, so it is arbitrarily shown as numerical; (3) the spacers are hypothetical, as usually a maximum of 6 nuclei were included in an isolate.

We are not the first to propose HGM in *Armillaria*. Ullrich and Anderson^19^ considered stable diploidy as the most likely explanation for prototrophy in mated auxotrophs of *Armillaria mellea*. However, they also presented an alternative hypothesis that they considered a less likely but possible explanation for their results: “Alternatively, it is possible that an unusual (unprecedented) type of heterokaryon is present, i.e*.,* one that is vegetatively stable in a filamentous fungus with uninucleate cells and intact septa.” Our results appear to be an example of Ullrich and Anderson’s alternative model.

**Figure 7:**
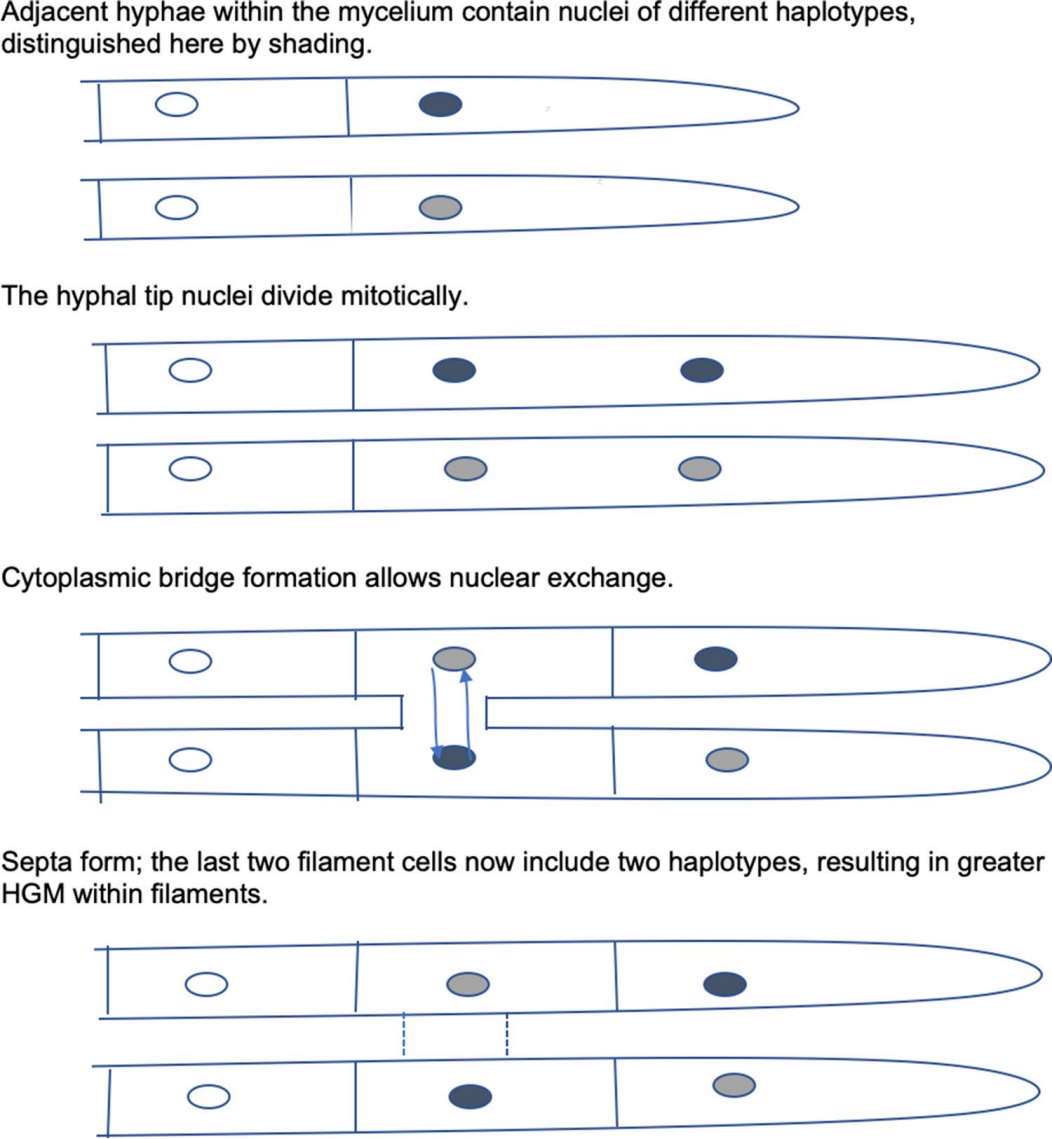
In this model, Haploid Genetic Mosaicism is maintained by nuclear exchange across cytoplasmic bridges connecting rhizomorph hyphal filament tips.

Because hyphal extension requires mitosis, contiguous compartments within growing hyphal tips should contain a series of identical nuclei. How then, in rhizomorphs capable of undergoing mitosis for decades, can within-hyphal filament HGM persist? Korhonen^20^ was the first to document nuclear migration through cytoplasmic bridges in *Armillaria*. We found cytoplasmic bridges to be common in monokaryotic rhizomorph hyphae collected in nature (Fig. 3) and hyphae grown in culture (Fig. 4). Because nuclei were frequently found in or near bridges, we propose nuclear exchange through bridges as a mechanism that maintains within-line and among-line HGM (Fig. 7).

#### Growth

Gallic acid growth experiments revealed significant line effects, treatment effects, and line × treatment effects for all 4 sets of Raynham and Bridgewater cell-lines (ANOVA *P* < 0.0001, Fig. 5, Supplementary Table S6). Because spores and rhizomorphs each possess genetic variation for growth and phenotypic plasticity, selection has the potential to affect their growth and phenotypic plasticity in nature.

Although reaction-norm shapes are similar in all four sets of Fig. 5 curves, the vertical spread of curves appears to be greater for spores than for rhizomorphs, and a posteriori paired t-tests show this is true at all 8 gallic acid concentrations (Supplementary Table S5). Variance differences could reflect different selection histories. *Armillaria gallica* spores disperse over long distances of up to 2 km^13^ and have the potential to land near hosts that produce very different concentrations of gallic acid. Selection in spores may therefore favor wide ranges of abilities to grow in the presence of gallic acid. In contrast, rhizomorphs assimilate nutrients and grow for long periods through soils and near hosts where conditions are comparatively stable. Environmental stability may have given rhizomorph genets in Bridgewater and Raynham opportunities to approach local adaptive norms. The immortal-strand^21^ hypothesis posits that asymmetric cell divisions preserve template DNA strands within stem cells. The dancing-genome hypothesis^2^ proposes that fungal nuclei can be distributed non-randomly to daughter cells in some fungi. Taken together these models suggest mechanisms that could maintain different populations of alleles in spore vs. rhizomorph stages of the life cycle. It would be interesting to see whether a priori comparisons of variances in other genets support this hypothesis.

#### Phenotypic plasticity

Cytoplasmic bridges permit exchanges of nuclei, nutrients, and other materials among hyphae^20,22–25^. Every intersection between graph lines in Fig. 5 represents a reversal in relative growth of 2 rhizomorph lines grown on media containing different gallic acid concentrations. In the Raynham rhizomorph graph, there are at least 70 intersections. Consider the intersection of Raynham rhizomorph hyphal filament lines r12 and r13 between gallic acid concentrations of 8 mM and 16 mM. In the lab, rhizomorph line r12 grows larger than line r13 on 8 mM media; r13 grows larger than r12 on 16 mM media (95% CIs do not overlap). In nature, if a rhizomorph containing hyphal lines r12 and r13 extends between areas where one host produces lower (8 mM) concentrations of gallic acid and another host produces higher (16 mM) concentrations, cytoplasmic bridges might favor movement of nuclei (and potentially nutrients) in one direction near the first host and in the opposite direction near the second host.

In other fungi, nuclei travel considerable distances along hyphal filaments^26,27^. If migrating nuclei in *A. gallica* cross cytoplasmic bridges, enter different hyphae, and undergo differential rates of mitosis, this process might create new populations of interacting haplotypes better suited to overcoming host defenses or utilizing resources of different hosts. The resulting reversible partitioning of nuclei among interconnected cells could help mosaic individuals deal with exposure to diverse environments over their long lives. This within-generation process could be seen as effectively equivalent to adaptive evolution that usually takes place between generations in species with unitary individuals. Environmentally-dependent synergisms within mycelia might also apply to interactions between mycorrhizal fungi and plants^28^, and therefore contribute to phenotypes that impart selective advantages in other types of haploid genetic mosaic organisms^29,30^. If organisms other than *A. gallica* respond to environmental conditions in this way, haploid genetic mosaics may be more common than is currently thought.

### Growth-study evidence of rhizomorph within-line genetic variance

ANOVA analyses of gallic acid growth trials suggest spores lack a within-line variance component that rhizomorphs possess. When averaged over 8 gallic acid concentrations, among-line variance accounts for an average of 95% of all spore-line growth variance (Table 2). Spore residual terms are presumed to be low (average = 5%) because they are affected by only plate-to-plate environmental variation. This is expected, given that spore mycelia lack cytoplasmic bridge connections among hyphae; and *EF1α* sequences show that spore within-line haploid genetic mosaic variation approaches zero. In contrast, rhizomorph residual terms (average = 19%) are approximately 4 times higher than spore residual terms (average = 5%) even though in comparison to spores their experimental plates are not expected to have higher levels of plate-to-plate environmental variation. We propose instead that rhizomorph residual terms are higher because they include a within-line genetic variance component that spores lack. Genetic variance is expected within rhizomorph hyphal filaments because they typically have multiple, among-hyphae cytoplasmic-bridge connections and have been shown to possess as many as 4 different *EF1α* sequences within individual hyphal filaments.

**Table 2:** On gallic acid media, among-line variance accounts for higher percentages of total growth variance in mycelia grown from spores than in mycelia grown from rhizomorphs.

Source	Genet	Number of experimental plates	Growth variance accounted for by among-line variance (%)	Residual term (%)
Spores	Raynham	392^a^	96	4
Spores	Bridgewater	396^a^	94	6
Rhizomorphs	Raynham	375^a^	77	23
Rhizomorphs	Bridgewater	398^a^	85	15

^a^Totals are lower than 400 as described in “Methods” section because 39 plates were contaminated (Supplementary methods).

### Haploid genetic mosaic organisms vs. diploid or haploid organisms

Because diploids have more mutation targets than haploids, they may have advantages in environments where adaptation is limited by total genetic diversity^3^. Haploid genetic mosaic *A. gallica* individuals, however, potentially have even more mutation targets than diploids; and differential nuclear replication and migration within individuals may allow beneficial alleles to increase in frequency within as well as between generations. Selection may eliminate harmful alleles more efficiently in haploids because their fitness effects are not masked. Haploid genetic mosaic *A. gallica* individuals may have advantages over strict haploids though, because nutrient flow within and among hyphal filaments may temporarily protect nuclei containing deleterious alleles so that they will remain available to be selected for in the event that environmental conditions change. The extreme longevity and size some *A. gallica* individuals attain suggest that life cycle features allow them to adapt to a wide range of environmental conditions over time and space, and we propose HGM may have contributed to this success.

## Methods

### Collection and somatic incompatibility testing

**Figure 8:**
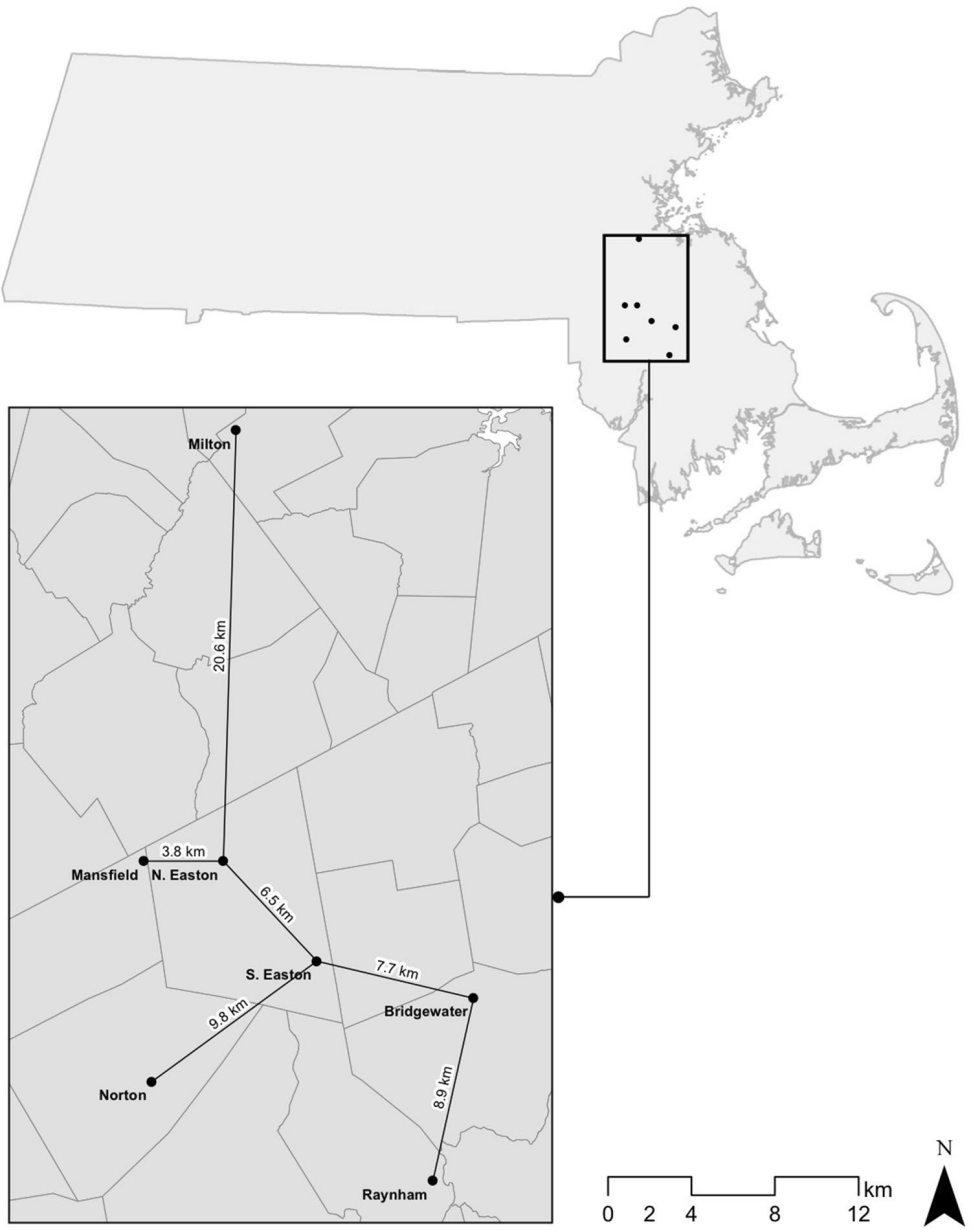
The samples collected from all seven sites in southeastern Massachusetts, USA, were shown by somatic incompatibility tests to represent different genets (individuals). (This map was created using ArcMap 10.4 with data from the Massachusetts Bureau of Geographic Information).

Spore cell lines, stipe hyphal filament lines, and rhizomorph hyphal filament lines used in this study were collected at the seven sites, separated by 3.8 to 20.6 km, shown in Fig. 8. Somatic incompatibility testing using a modified Shaw/Roth medium^14^ confirmed that each of these collections represents a distinct genet.

### Cell line and hyphal filament line isolation

Spore cell lines and stipe hyphal tip lines were isolated according to published methods^7,17^; modified versions of these methods were used to isolate rhizomorph and soil mycelium hyphal filament lines (Supplementary methods). Based on 95 length measurements of rhizomorph hyphal compartments and the inside diameter of the Pasteur pipettes used to excise them, we estimate that, on average, six contiguous hyphal compartments were harvested each time this method was used to isolate a hyphal filament line.

### Microspectrophotometry for ploidy evaluation

Nuclear DNA content was measured in Bridgewater and Raynham genets using DAPI (4′,6-diamidino-2-phenylindole)-DNA staining and microspectrophotometric methods^17,18^. For purposes of comparison, spore nuclei and prophase I basidial nuclei, each fixed in 95% ethanol within 2 h of collection from nature, were used as standards to establish expected quantities of DAPI-stained DNA in haploid and diploid cells respectively. The use of spores as haploid standards was supported by Baumgartner’s independent analyses of banding patterns in 4 polymorphic microsatellite loci and 2 polymorphic nuclear loci of Raynham spore samples (personal communication). Because DAPI-DNA values are expressed in arbitrary units (a.u.), values for spore nuclei and prophase I basidial nuclei in the current study were normalized with values reported earlier^8^.

### RFLP analyses

RFLP patterns were resolved for five DNA regions at all seven collection sites. DNA was isolated from hyphae removed from mycelia grown for three weeks at 23 °C on cellophane placed on 3% MEA. ZR Fungal/Bacterial DNA Isolation Kits (Zymo Research) were used to isolate genomic DNA from rhizomorph hyphae, stipe hyphae, and hyphae grown from individual spores. PCR products were digested with restriction endonucleases (RE) according to manufacturer’s instructions (reaction mixtures contained 10 µL recommended 2 × NEB buffer, 8 µL PCR product, and 2 µL RE). *G3PDH* was digested with *RsaI*, *EF1α* and *IGS1* with *HaeIII*, *DP2* with *BstUI*, and *DP5* with *DdeI*. PCR parameters are described in Supplementary methods. RE digested samples were run on 3% agarose gels containing ethidium bromide (loaded with 10 µL RE digest and 2 µL 6 × loading-tracking dye).

### *EF1α* sequences

*EF1α* sequences were determined for four of the seven collection sites (Raynham, N. Easton, Norton, and Milton). The genomic DNA that was isolated for RFLP analyses was used also for cloning. PCR parameters are described in Supplementary methods. The size of products was determined by electrophoresis on a 1% agarose gel containing ethidium bromide. Bands of the appropriate size were cut from gels and purified using a QIAquick Gel Extraction Kit (Qiagen) following the manufacturer’s protocol but replacing Buffer EB with 18 MΩ water (Elga). The resulting EF1α amplicons were ligated into vectors using a Zero Blunt TOPO PCR Cloning Kit (Thermo Scientific). Competent NEB 5α *E. coli* High Efficiency cells were then transformed with the recombinant DNA and incubated on LB-kanamycin agar plates for 24–28 h in a 37 °C incubator. Plasmid insert size was checked with colony PCR using M13 primers and Taq Master Mix (NEB). Colonies with inserts of the correct size were used to inoculate LB-kanamycin broths and then incubated 12–16 h in a 37 °C shaker incubator. Plasmid DNA was then isolated from the bacteria using the Spin Miniprep of Plasmid DNA kit (Qiagen) and sequenced at Tufts Core Facility. Sequences were analyzed using online BLAST (NCBI) and aligned using Clustal W; and restriction sites were verified using NEB Cutter to allow comparison of sequences with RFLP data.

### Examination of mycelia for evidence of cytoplasmic bridges

Cultured spore cell lines, stipe hyphal filament lines, rhizomorph hyphal filament lines, and spore, stipe, and rhizomorph samples fixed in 95% ethanol within 2 h of collection from nature were stained with DAPI^17,18^. Samples were initially scanned for the presence of cytoplasmic bridges connecting adjacent hyphae using phase contrast microscopy and then examined for the presence of DAPI-stained nuclei near or in cytoplasmic bridges using epifluorescence.

### Growth study media

Bark-extract vs. wood-extract growth media preparation is explained in Supplementary methods. In the gallic acid growth study, in order to include and exceed the range of gallic acid concentrations we have measured, or that have been published for likely *Armillaria* hosts^31–39^, media were prepared with concentrations of 0, 4, 8, 16, 24, 32, 40, and 48 mM gallic acid. To control for pH, enough 1 M NaOH (or 1 M HCl) was added to adjust the pH of all media to 4.5, a value based on an earlier published study^40^. To control for water potential effects of gallic acid and NaOH (or HCl), enough KCl was added to adjust the water potential of all media to the same value. After media were prepared, a Wescor Dew Point Microvoltmeter (model HR-33T) was used to measure actual water potential values for liquid components of all eight media; measured water potentials of liquid components (before malt extract and agar were added) ranged from − 2.48 to − 2.44 MPa (mean =  − 2.47, standard deviation = 0.02); and estimated water potentials of all components including malt extract and agar ranged from − 3.35 to − 3.30 (mean =  − 3.33, standard deviation = 0.02). The water potential of − 3.33 MPa was based on a value published earlier^8^. Separate experiments were conducted with spore cell lines and rhizomorph hyphal tip lines to determine whether a choice of KCl, NaCl, or sucrose as osmotica would affect growth. These tests demonstrated that the choice of osmoticum had no effect on growth of either rhizomorph hyphal filament lines (ANOVA *P* = 0.7483) or spore cell lines (ANOVA *P* = 0.4147), confirming an earlier report^41^.

### Growth study experimental design

For simplicity, in this experimental design section and in the statistical analyses section, the phrases “cell line” and “hyphal filament line” will both be referred to as “line.” The crude bark-extract/wood-extract experiment and the gallic acid experiment were each designed to detect three effects: line effects, treatment effects, and line × treatment effects. The bark-extract/wood extract design details are described in Supplementary methods. In the gallic acid experiment, the basic experimental unit was an array of 50 plates consisting of five replicate plates for each of ten different lines. Separate 50-plate arrays were exposed to eight different concentrations of gallic acid. The experiment therefore included eight 50-plate arrays of *A. gallica* spore lines from Bridgewater, eight 50-plate arrays of *A. gallica* rhizomorph lines from Bridgewater, eight 50-plate arrays of *A. gallica* spore lines from Raynham, and eight 50-plate arrays of *A. gallica* rhizomorph lines from Raynham for a total of 1600 experimental plates. Additional details of experimental design are described in Supplementary methods.

### Statistical analyses

Nuclear DAPI-DNA values of prophase I basidia and spores were compared with a t-test. Two-way mixed-model ANOVA (line = random factor, treatment = fixed factor) was used to test for: (i) *line effect* which tests for among-line heritable phenotypic variation in growth; (ii) *treatment effect* which tests for the effect of treatments (i.e., either bark vs. wood or gallic-acid concentration); and (iii) *line* × *treatment effect* which tests for among-line heritable phenotypic variation in response to treatment (i.e., either bark vs. wood or gallic-acid concentration). Paired t-tests were used to compare variances because each spore line variance calculated at a given gallic acid concentration had a corresponding rhizomorph line variance that was calculated at the same gallic acid concentration. All statistical computations were carried out with JMP Pro 12 or 14.

## Supplementary information

Supplementary Information.

## Data Availability

The 190 EF1α sequences generated and analyzed during this study are available in GenBank, accession numbers MW025276-MW025465.
