# Supplementary material for: Mosaic fungal individuals have the potential to evolve within a single generation
Source: Sci Rep. 2020 Oct 19;10:17625. doi: 10.1038/s41598-020-74679-5 (PMC7572425; doi:10.1038/s41598-020-74679-5)
Supplement: Supplementary file 1 — Supplementary Information. [file 41598_2020_74679_MOESM1_ESM.pdf]

Mosaic fungal individuals have the potential to evolve within a single generation

Maura G. Tyrrell[1*], Diane C. Peabody[1], Robert B. Peabody[1], Magdalena James-Pederson[1], Rachel G. Hirst[1], Elisha Allan-Perkins[1], Heather Bickford[1], Amy Shafrir[1], Robert J. Doiron[1], Amber C. Churchill[1], Juan Carlos Ramirez-Tapia[1], Benjamin Seidel[1], Lynes Torres[1], Kathryn Fallavollita[1], Thomas Hernon[1], Lindsay Wiswell[1], Sarah Wilson[1], Erica Mondo[1], Kathleen Salisbury[1], Carrie Peabody[1], Patrick Cabral[1], Lauren Presti[1], Kelsey McKenna-Hoffman[1], Michele Flannery[1], Kaitlin Daly[1], Darius Haghighat[1], Daniel Lukason[1]

[1]Biology Department, Stonehill College, Easton, MA, USA 02357

*Correspondence to mtyrrell@stonehill.edu

Table of contents

Supplementary Data

Supplementary Table S1. Haploidy of spore, rhizomorph, and soil mycelia nuclei 3

Supplementary Table S2. RFLP patterns for seven genets 4

Supplementary Table S3. EF1α haplotypes of spores and rhizomorphs from Norton, N. Easton, and Milton genets.. 5-7

Supplementary Table S4. Bark and wood growth ANOVA’s..8

Supplementary Figure S1. Growth of spore- and rhizomorph-derived mycelia on bark and wood extracts 9

Supplementary Table S5. Variances for spore- and rhizomorph-derived mycelia grown on media with gallic acid 10

Supplementary Table S6. ANOVA’s for spore- and rhizomorph-derived mycelia grown on media with gallic acid 11

Supplementary Methods

Isolation of rhizomorph and soil mycelium hyphal filament lines 12

PCR parameters for RFLP analysis 13

PCR parameters for EF1α sequence analysis 14

Bark-extract vs. wood-extract media preparation..14

Bark-extract vs. wood-extract and gallic acid growth studies experimental design......................15

Supplementary Table S1. DAPI-DNA analysis shows Prophase I basidial nuclei have more DNA than spore, rhizomorph, or soil mycelial nuclei. Prophase 1 nuclei are diploid; spore, rhizomorph and soil mycelia nuclei are all haploid.

Sample source	***N***	***Median nuclear DNA in arbitrary units (a.u.)***	***Mean nuclear DNA a.u.± 1 s.d.***	***Presumed nuclear condition***
prophase I basidia (field-collected)[a]	30	216	219 ±31 [c]	Because prophase I nuclei are known to be diploid and replicated, 216 and 219 a.u. establish the value for 4C.
spores (field-collected)[a]	30	87	83 ±22 [c]	87 and 83 a.u. are consistent with a mixture of 1C (unreplicated) and 2C (replicated) haploid nuclei.
rhizomorphs and soil mycelia (field-collected)[a]	50	77	84 ± 31	77 and 84 a.u. are consistent with a mixture of 1C (unreplicated) and 2C (replicated) haploid nuclei.
rhizomorphs (cultured)[b]	202	79	83 ± 22	79 and 83 a.u. are consistent with a mixture of 1C (unreplicated) and 2C (replicated) haploid nuclei.

[a] "field-collected" denotes samples fixed in 95% ethanol within 2 hours of collection from nature.
[b] "cultured" denotes samples grown in culture before fixation in 95% ethanol.
[c] Prophase I basidia nuclei had significantly more DNA than spore nuclei (t-test; $P < 0.0001$).

Supplementary Table S2. RFLP patterns for seven genets show only single haplotypes for spore-derived mycelia but many combination patterns for mycelia grown from stipes and rhizomorphs.

			DNA region				
**genet**	**source**	**N**	**G3PDH × RsaI**	**EF1α × HaeIII**	**IGS1 × HaeIII**	**DP2 × BstUI**	**DP5 × DdeI**
Milton	spore	10	R1[a]	E2 or E3	H1	B1	D2
	stipe	10	R1	**E2E3**	H1	B1	D2
	rhizomorph	10	R1	**E2E3**	H1	B1	D2
Mansfield	spore	10	R2	E3	H1	B1 *or* B2	D2
	stipe	10	R2	E3	H1	**B1B2**	D2
	rhizomorph	10	R2	E3	H1	**B1B2**	D2
N. Easton	spore	10	R2	---	H3	B3	D2
	stipe	10	R2	---	H3	B3	D2
	rhizomorph	10	R2	**E3E4**	**H1H2**	**B1B2**	D2
S. Easton	spore	10	R2	---	H3	B1 *or* B3	---
	stipe	10	R2	---	H3	**B1B3**	---
	rhizomorph	10	R2 *or* R3	---	H3	**B1B3**	---
Norton	spore	10	R2	---	H3	B3	---
	stipe	10	R2	---	H3	B3	---
	rhizomorph	10	**R1R2**	E3	**H1H2**	**B1B2**	---
Bridgewater	spore	10	R1 *or* R2	E2 *or* E3	H1 *or* H2	B1 *or* B2	D1 *or* D2
	stipe	10	R2 *or* **R1R2**	E2 *or* E3	H1 *or* H2	**B1B2**	D2 *or* **D1D2**
	rhizomorph	10	**R1R2**	E3	H1	**B1B2**	D2 *or* **D1D2**
Raynham	spore	10	R1 *or* R2	E1 *or* E2	H1 *or* H2	B1 *or* B2	D1
	stipe	10	**R1R2**	**E1E2**	---	**B1B2**	**D1D2**
	rhizomorph	10	**R1R2**	**E1E2**	**H1H2**	**B1B2**	**D1D2**

[a]RFLP letter-number notations represent presumed alleles with the fragment size distributions listed below. Twenty-seven bolded notations, seen only for stipes or rhizomorphs, indicate RFLP patterns that combine two alleles. Three underlined notations show vegetative stages with polymorphic allele patterns similar to those of polymorphic spores.

Fragment size distributions

R1 = 354, 188, 114, 9	E1 = 309, 274, 150	H1 = 316, 211, 164, 84, 55, 30, 27, 10	B1 = 500, 168, 132	D1 = 145, 126, 119
R2 = 543, 114, 9	E2 = 234, 162, 150, 147, 40	H2 = 316, 211, 191, 84, 55, 30, 10	B2 = 263, 237, 168, 132	D2 = 264, 126
R3 = 303, 165, 114, 75, 9	E3 = 274, 162, 150, 147	H3 = 316, 211, 191, 114, 55, 10	B3 = 431, 237,132	
	E4 = 309, 234, 150, 40			

Total PCR product size (bp)

665/666	733	897	800	390

Supplementary Table S3. *EF1α* haplotypes of rhizomorphs are more variable than those of spores in the Norton, Easton, and Milton genets. All SNP's differing from those of haplotype 1 are shaded and the count is shown in column 2 (Δ). For each clone, the notation sequence is source (s for spore or r for rhizomorph), then cell or hyphal filament line number, then clone number after the hyphen. (a) Norton, (b) N. Easton, and (c) Milton

a. Norton

haplotypes	Δ	SNP position* 353	360	394	632	700	747	**spore clones**	**rhizomorph clones**	**#spore clones**	**#rhizo. clones**	**#spore lines**	**#rhizo. lines**
1	-	C	C	G	A	T	A	**s1-1 s2-1 s2-2 s4-1 s4-2 s5-1 s6-2 s9-1 s9-2**		9		6	
**2**	**1**	**C**	**C**	**A**	**A**	**T**	**A**	**s16-1 s16-2 s16-1' s16-2' s8-3 s15-1 s15-2**		**7**		**3**	
3	2	C	C	G	C	T	C		r3-2 r3-4 r3-10 r8-3 r8-4 r8-8 r9-9		7		3
4	2	C	C	G	C	T	T		r31-4 r2-1 r2-3 r3-1 r3-3 r8-6 r8-9 r9-4 r9-10		9		5
5	2	C	C	G	T	T	T		r13-3 r10-1 r10-3 r10-4 r20-3 r20-5		6		3
6	2	C	C	G	T	T	C		r29-5		1		1
7	3	C	C	G	T	C	T		r13-4		1		1
8	3	C	T	G	C	T	C		r7-1 r7-3 r7-4 r7-5 r7-6 r7-7 r7-8		7		1
9	3	A	C	G	C	T	C		r31-5 r2-5 r3-8 r3-9 r8-7 r8-10 r9-6 r7-2		8		6
10	3	A	C	G	C	T	T		r31-1 r2-2 r2-4 r3-5 r3-7		5		3
11	3	A	C	G	T	T	C		r13-2 r10-5 r20-2 r29-4		4		4
										**16**	**48**	**9**	**10****

*Numbering is from the start of the amplified region.
**Some rhizomorph hyphal tip lines have multiple SNP haplotypes: r7, r10, r20, and r29 have two; r2, r8, r9, r13, and r31 have three; r3 has four. No spore cell lines have multiple SNP haplotypes.

b. N. Easton

haplotypes	D	44	53	59	71	83	86	92	107	125	160	167	168	171	175	180	182	182'	183	188	196	202	236	269	287	290	338	353	354	354'	369	377	378	390	392	398	446	599	614	617	629	632	719	725	735	736	747	751	763	spore clones	rhizomorph clones	# spore clones	# rhizo. clones	# spore lines	# rhizo. lines
1	-	A	T	T	C	G	T	C	T	C	A	A	C	T	A	T	T	C	C	G	G	C	C	T	T	C	A	C	C	C	C	A	G	C	C	C	C	C		C	C	A	C	G	A	T	A	G	G	**s1-1 s1-2**		2		1	
2	*1*	A	T	T	C	G	T	C	T	C	A	A	C	T	A	T	T	C	C	G	G	C	C	T	T	C	A	C	C	C	C	A	G	C	C	C	C	C	C	C	C	A	C	G	A	T	A	G	G	**s16-1 s19-1 s19-2**		3		2	
3	*2*	A	T	T	C	G	T	C	C	C	A	A	C	T	A	T	T	C	C	G	G	C	C	T	T	C	A	C	C	C	C	A	G	C	C	C	C	C	C	C	C	A	C	G	A	T	A	G	G	**s16-2**		1		1	
4	*44*	G	C	C	T	A	C	C	C	T	G	G	T	C	G	G	G	-	T	C	A	T	T	C	C	T	G	C	T	-	T	G	C	T	T	C	T	T	C	C	T	C	T	A	C	G	C	T	A		r13-2		1		1
5	*44*	G	T	C	T	A	C	T	C	T	G	G	T	C	G	G	G	-	T	C	A	T	T	C	C	T	G	C	T	-	T	G	C	T	T	C	T	T	C	C	T	C	T	A	C	G	C	T	A		r13-3 r13-4		3		1
6	*45*	G	T	C	T	A	C	T	C	T	G	G	T	C	G	G	G	-	T	C	A	T	T	C	C	T	G	C	T	-	T	G	C	T	T	T	T	C	C	T	T	C	T	A	C	G	C	T	A		r16-7		1		1
7	*45*	G	T	C	T	A	C	C	C	T	G	G	T	C	G	G	G	-	T	C	A	T	T	C	C	T	G	A	T	-	T	G	C	T	T	T	T	C	C	T	T	C	T	A	C	G	C	T	A		r4-2		1		1
8	*45*	G	T	C	T	A	C	T	C	T	G	G	T	C	G	G	G	-	T	C	A	T	T	C	C	T	G	C	T	-	T	G	C	T	T	C	T	T	C	T	T	C	T	A	C	G	C	T	A		r4-1 r4-3 r16-6 r33-7		4		3
9	*45*	G	C	C	T	A	C	C	C	T	G	G	T	C	G	G	T	-	T	C	A	T	T	C	C	T	G	A	T	-	T	G	C	T	T	T	T	C	C	T	T	C	T	A	C	G	C	T	A		r11-2		1		1
10	*45*	G	C	C	T	A	C	C	C	T	G	G	T	C	G	G	G	-	T	C	A	T	T	C	C	T	G	C	T	-	T	G	C	T	T	C	T	T	C	T	T	C	T	A	C	G	C	T	A		r5-3 r5-4		2		1
11	*46*	G	C	C	T	A	C	C	C	T	G	G	T	C	G	G	G	-	T	C	A	T	T	C	C	T	G	A	T	-	T	G	C	T	T	T	T	C	C	T	T	C	T	A	C	G	C	T	A		r1-1 r5-2 r5-5 r17-6 r17-8 r33-8		6		4
12	*46*	G	T	C	T	A	C	T	C	T	G	G	T	C	G	G	G	-	T	C	A	T	T	C	C	T	G	A	T	-	T	G	C	T	T	T	T	C	C	T	T	C	T	A	C	G	C	T	A		r1-2 r33-6		2		2
																																																				**6**	**21**	**3****	**8****

*Numbering is from the start of the amplified region.
**Some have multiple SNP patterns.

c. Milton

SNP position*

haplotypes	D	23	50	53	77	168	180	389	390	446	566	614	629	668	695	719	722	726	736	745	747	751	756	763	spore clones	rhizomorph clones	# spore clones	# rhizo. clones	# spore lines	# rhizo. lines
1	--	C	G	C	C	C	T	T	C	C	T	C	C	A	T	C	A	G	A	C	A	G	T	G	**s1-1, s6-2, s21-3, s28-1, s28-2, s28-3, s33-1, s33-2, s33-3, s36-1, s36-2, s36-3**		12	0	6	0
2	1	C	G	C	C	C	T	T	C	C	T	C	C	A	T	C	C	G	A	C	A	G	T	G		r1-1, r1-3, r2-2, r3-1, r3-2, r4-1, r4-2, r5-2, r6-3, r8-1, r9-1, r26-2	0	12	0	9
3	5	C	G	C	C	C	T	T	C	T	C	C	T	G	T	C	C	-	A	C	A	G	T	G		r7-1	0	1	0	1
4	6	C	G	C	C	C	T	T	C	C	T	C	C	A	T	C	C	G	A	T	C	T	C	A		r5-3	0	1	0	1
5	13	T	A	T	T	T	G	T	C	T	C	T	T	G	C	C	C	G	A	C	A	G	T	G		r4-3	0	1	0	1
6	14	C	G	C	C	C	T	T	C	T	C	C	T	G	C	T	T	A	G	T	C	T	C	A		r9-3	0	1	0	1
7	14	T	A	T	C	T	G	G	T	T	C	T	T	G	C	C	C	G	A	C	A	G	T	G		r6-2	0	1	0	1
8	15	C	G	T	C	C	T	T	C	T	C	C	T	G	C	T	T	A	G	T	C	T	C	A		r1-2	0	1	0	1
9	19	C	A	C	C	T	G	G	T	T	C	C	T	G	C	T	T	A	G	T	C	T	C	A		r8-3	0	1	0	1
10	21	C	A	T	C	T	G	G	T	T	C	T	T	G	C	T	T	A	G	T	C	T	C	A		r26-1	0	1	0	1
11	22	T	A	C	T	T	G	G	T	T	C	T	T	G	C	T	G	A	G	T	C	T	C	A	**s6-1, s6-3, s8-1, s8-2, s8-3, s19-1, s19-2, s19-3, s25-1, s25-2, s25-3**		11	0	4	0
12	23	T	A	T	T	T	G	G	T	T	C	T	T	G	C	T	T	A	G	T	C	T	C	A		r2-3, r6-1	0	2	0	2
																											**23**	**22**	**9****	**10****

*Numbering is from the start of the amplified region.
**Some have multiple SNP patterns.

Supplementary Table S4. Mycelia areas differ significantly for almost all soil mycelium hyphal filament lines and spore cell lines grown on bark or wood extracts of four tree species. Two-way, mixed model ANOVA (line = random effect, treatment = fixed effect). N = 1,600 culture plates, 1590 with independent environmental histories (Supplementary Methods)

	Effect	*F-value*	*DF*	*P-value*[a]
**Spore lines growing on…**				
P. strobus	line	28.594	9	< 0.0001
	treatment	0.384	1	0.5362
	line × treatment	3.352	9	0.0008
T. canadensis	line	21.009	9	< 0.0001
	treatment	18.002	1	< 0.0001
	line × treatment	3.742	9	0.0003
Q. alba	line	18.707	9	< 0.0001
	treatment	315.728	1	< 0.0001
	line × treatment	5.557	9	< 0.0001
Q. rubra	line	23.201	9	< 0.0001
	treatment	1386.318	1	< 0.0001
	line × treatment	15.167	9	< 0.0001
**Soil mycelia lines growing on…**				
P. strobus	line	14.911	9	< 0.0001
	treatment	12.492	1	0.0005
	line × treatment	1.297	9	0.2413
T. canadensis	line	11.216	9	< 0.0001
	treatment	67.742	1	< 0.0001
	line × treatment	4.648	9	< 0.0001
Q. alba	line	8.696	9	< 0.0001
	treatment	3.577	1	0.0602
	line × treatment	5.020	9	< 0.0001
Q. rubra	line	5.307	9	< 0.0001
	treatment	37.373	1	< 0.0001
	line × treatment	6.083	9	< 0.0001

Supplementary Figure S1. On red oak extracts, six of 10 soil mycelium hyphal tip lines grew larger on bark extracts than on wood extracts; all 10 spore cell lines grew larger on wood extracts than on bark extracts. Two-way, mixed model ANOVA (line = random effect, treatment = fixed effect (** $P < 0.01$; *** $P < 0.001$). Area units are mean mycelial areas after 21 days for 10 replicates of each cell line. Bark: ▯ Wood: ▯ (N = 397 culture plates, 394 with independent environmental histories. (Supplementary Methods)
.

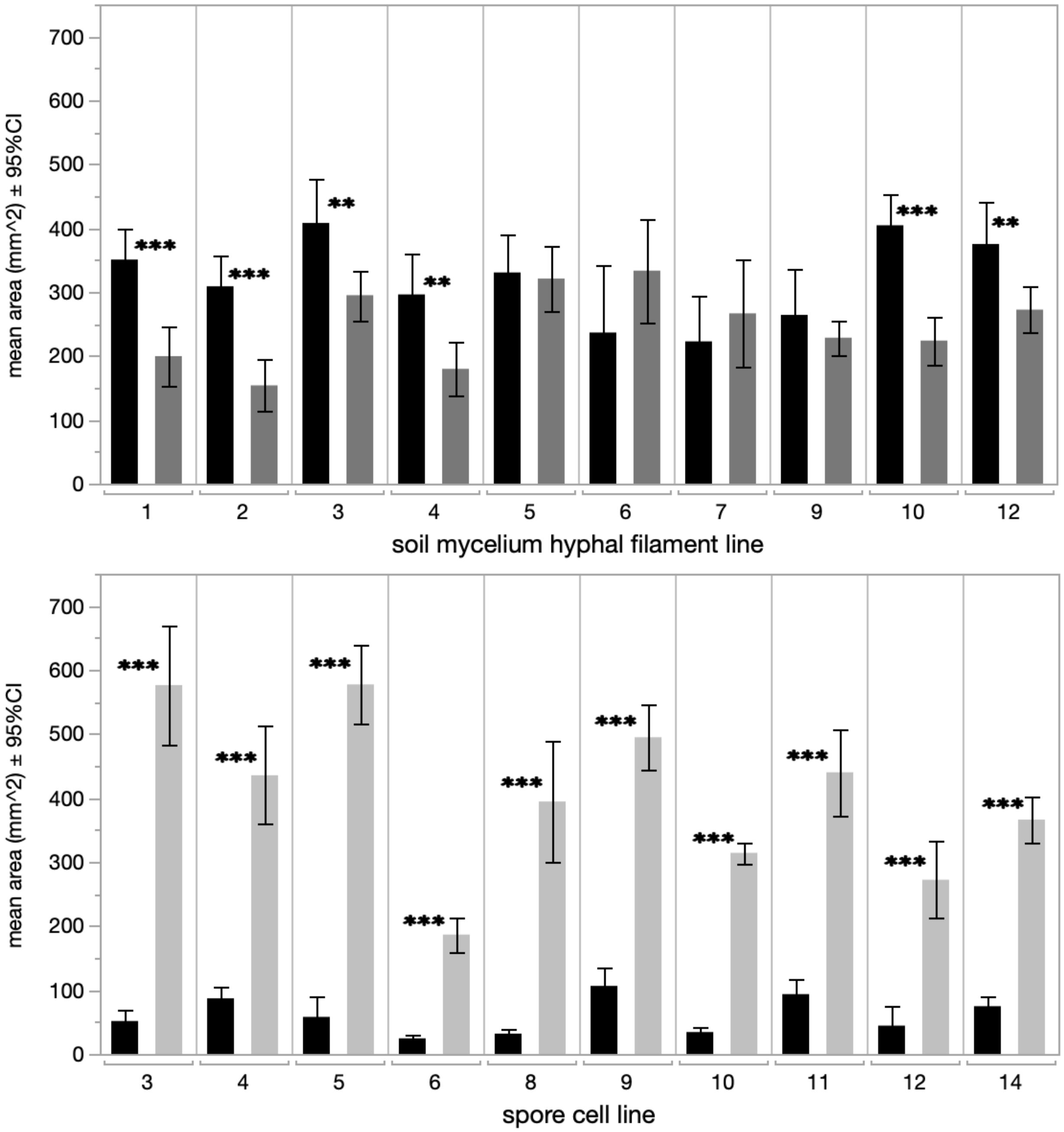

Supplementary Table S5. Variance in growth was greater for spore cell lines than for rhizomorph hyphal filament lines in (A) Bridgewater (paired t-test = 9.229, $P < 0.0001$) and (B) Raynham (paired t-test = 4.263, $P = 0.0037$).

**A. Bridgewater genet**	***Variances in growth (as areas after 21 days)***	
Gallic acid (mM)	***Spore cell lines***	***Rhizomorph hyphal tip lines***
0	36572	18569
4	52534	45418
8	56244	38107
16	54467	35133
24	47056	20361
32	33072	8912
40	29948	5391
48	27024	3784
**mean**	**42115**	**21959**

**B. Raynham genet**	***Variances in growth (as areas after 21 days)***	
Gallic acid (mM)	***Spore cell lines***	***Rhizomorph hyphal tip lines***
0	22615	18353
4	27826	10782
8	26449	4851
16	20876	5168
24	16853	4308
32	19657	2770
40	5524	1537
48	4601	2227
**mean**	**16925**	**6250**

Supplementary Table S6. Reaction norms for Bridgewater and Raynham genets show significant variation among cell lines for both growth and phenotypic plasticity in response to gallic acid concentration (=line x [gallic acid]). Two-way, mixed model ANOVA (line = random effect, treatment = fixed effect).

Genet/cell type	***Effect***	***F-value***	***DF***	***P-value***
Raynham spore lines	line	859.855	9	< 0.0001
	[gallic acid]	276.183	7	< 0.0001
	line × [gallic acid]	19.457	63	< 0.0001
Raynham rhizomorph lines	line	97.698	9	< 0.0001
	[gallic acid]	322.156	7	< 0.0001
	line × [gallic acid]	9.119	63	< 0.0001
Bridgewater spore lines	line	610.429	9	< 0.0001
	[gallic acid]	224.981	7	< 0.0001
	line × [gallic acid]	4.291	63	< 0.0001
Bridgewater rhizomorph lines	line	205.349	9	< 0.0001
	[gallic acid]	166.977	7	< 0.0001
	line × [gallic acid]	6.744	63	< 0.0001

Supplementary methods

Isolation of rhizomorph and soil mycelium hyphal filament lines

To isolate rhizomorph hyphal filament lines, approximately 2.5 cm pieces of rhizomorphs were excised and scrubbed with a brush in tap water to remove soil and debris. Pieces were transferred to sterile water in a sterile Petri dish, soaked for 1 minute, and then blotted on sterile paper towels. Pieces were transferred to full strength bleach (6% hypochlorite solution) for 2.5 minutes, rinsed twice in sterile water, and blotted on sterile paper towels. Ends of rhizomorph pieces were removed and remaining portions cut into 2 mm pieces and placed on modified Worrall's BDS medium[31]. Rhizomorph pieces were checked for growth every 24 h; and when growth was observed at the ends a rhizomorph, the piece was moved to a new plate of modified BDS medium. Under an inverted microscope, the area from which the rhizomorph piece had been removed was checked for new growth. When we observed a hyphal tip growing at least 500 μm from other hyphal tips, we excised it using a bent sterile Pasteur pipette. The cylinder of medium containing the excised hyphal tip was placed on a plate of malt extract agar (MEA) and observed for new growth. Based on 95 length measurements of rhizomorph hyphal compartments and the inside diameter of a bent Pasteur pipette, we estimate that, on average, six contiguous hyphal compartments were harvested each time this method was used to isolate a hyphal tip. To isolate soil mycelium hyphal filament lines, hyphae were isolated from a strawberry plant acting as bait and growing in soil within 20 cm of a basidiome of *A. gallica* in Raynham, Massachusetts[9]. Roots and leaves of the strawberry plant were removed; the remaining stem was cut lengthwise into two pieces; and clumps of hyphae growing within the stem were placed on BDS medium. After 48 h, hyphal clumps that appeared to be uncontaminated were moved to MEA. After new growth was confirmed, a bent sterile Pasteur

pipette was used to excise a single hyphal tip as described above for rhizomorphs. Somatic incompatibility (SI) tests using a modified Shaw/Roth medium[14] confirmed that these soil mycelia belonged to the same genetic individual that produced the basidiome above (*i.e.*, the basidiome located 20 cm from the strawberry plant).

PCR parameters for RFLP analysis

Polymerase chain reaction (PCR) amplifications for five DNA regions (*DP2, DP5, EF1α, IGS1, G3PDH*) were carried out using an Applied Biosystems 2400 thermal cycler in 30 µL reactions containing 7.0 µL nuclease-free water, 1.5 µL forward primer, 1.5 µL reverse primer, 5.0 µL genomic undiluted genomic DNA, and 15.0 µL Phusion 2x Master Mix from New England Biolabs (NEB). Primers for *DP2* (Hodnett and Anderson, 2000) were DP2f: 5´-GTGAAGCTGCATTGGATAACC-3´ and DP2r: 5´-GGAAGGAACTCACCAAGTACG-3´ with an annealing temperature of 45 °C. Primers for *DP5* (Hodnett and Anderson, 2000) were DP5f: 5´-TGCAGAGAACCGTGAATTA-3´ and DP5r: 5´-ATTCGCAATTCTGCACGC-3´ with an annealing temperature of 54.3 °C. Primers for *EF1α* (Baumgartner et al. 2010) were EF-AMf: 5´-AGGCTGATTGTGCCATTCTC-3´ and EF-AM 785r: 5´-CCTTGACGGAGACGTTCCTA-3´ with an annealing temperature of 58 °C. Primers for *IGS1* (Harrington and Wingfield, 1995) were O-1: 5´-AGTCCTATGGCCGTGGAT-3´ and LR12R: 5´-CTGAACGCCTCTAAGTCAGAA-3´ with an annealing temperature of 60 °C. Primers for *G3PDH* (Baumgartner et al. 2012) were GPD1-Arm: 5´-GCCAAGAAGGTCGTCA TCTC-3´ and GPD2-Arm: 5´-AGTAACCCCACTCGTTGTCG-3´ with an annealing temperature of 59 °C. PCR cycling parameters for all five DNA regions were: 95 °C for 30 s, then 35 cycles of 95 °C for 30s, the annealing temperatures listed above for 40 s, and 68 °C for 1 m, followed by a

final extension at 68 °C for 5 m. Presence of genomic DNA and PCR products was confirmed on 1% agarose gels containing ethidium bromide (loaded with 5 µL PCR product, 5 µL water, and 2 µL 6X loading-tracking dye).

PCR parameters for EF1α sequence analysis

Primers EF-AMf and EF-AM785r were used in Phusion master mix to amplify a portion of the *EF1α* gene. For some samples, a modified touchdown PCR was used[1]. Touchdown PCR cycling parameters were: denaturation at 94°C for 2 m, then 10 cycles (94°C for 40 s, 60°C for 40 s [minus 1°C per cycle], then 72°C for 2 m), then 36 cycles (94°C for 45 s, 53° for 90 s, 72°C for 2 m), then 72°C for 10 m.

Bark-extract vs. wood-extract media preparation

Bark and wood samples collected in June from eastern white pine (*Pinus strobus*), eastern hemlock (*Tsuga canadensis*), white oak (*Quercus alba*), and northern red oak (*Quercus rubra*) were used to prepare eight different types of crude-extract media. Fresh samples of bark and sapwood were converted into sawdust with a clean rasp; sufficient MilliQ water (18.2 MΩ) was added to each sample to make a final concentration of 46.27 g sawdust (wood or bark)/L water. Hydrated samples were warmed to 37°C and soaked at 37°C for 24 hours; the resulting liquid from each sample was filtered four times through folded cheesecloth. After the bark and wood samples were filtered, they were brought up to their original volumes with MilliQ water. Filtrates were stored at 5°C until used to make agar media. Prior to autoclaving, 1 L of each filtrate was decanted into a 2-L flask containing 15 g agar. Autoclaved media were dispensed in 10 mL aliquots into 60 × 15 mm Petri dishes.

Bark- vs. wood-extract and gallic acid growth studies experimental design

In both experiments, inoculation of each replicate experimental plate from a separate inoculum plate with its own, independent environmental history, controlled for environmental history of replicate plates. Thus, in the bark-extract/wood-extract experiment, each plate in a 100-plate experimental array was inoculated from its own separate plate in a 100-plate inoculum plate array, each with its own, independent environmental history; and in the gallic acid experiment, each plate in a 50-plate, experimental array was inoculated from its own separate plate in a 50-plate inoculum plate array, each with its own, independent environmental history. Controlling for environmental history means that when greater phenotypic similarities are seen within lines than among lines, it is safe to conclude that these similarities are present because replicate plates within lines are genetically similar, and not because they came from a common inoculum plate where they shared a recent, common environmental history.

A perfect one-to-one correspondence between inoculum plates and experimental plates required that none of the plates in an inoculum-plate array became contaminated. To compensate for a small number of inoculum plates that did become contaminated, several uncontaminated inoculum plates were used as inoculum sources more than once. Of the 1600 plates in the bark-extract/wood-extract experiment, 1590 (99.4%) had independent environmental histories; of the 1600 plates prepared for the gallic acid experiment, only 1571 were inoculated because 29 plates were contaminated; and of these, 1547 (98.5%) had independent environmental histories.

Environmental-history lines (inoculum plates) were individually inoculated onto malt extract agar plates (1.5 % malt extract, 1.5% agar) with 5mm diameter agar plugs cut with a # 2 cork borer; plates were then placed in haphazard array with respect to vertical and horizontal positions in a dark 23 °C incubator for 27-28 days until they were used to inoculate experimental plates in the bark-extract/wood-extract experiment. For the experiment itself, plates were placed in a dark 23 °C incubator for 14 days. For the gallic acid experiment, similar procedures were used but inoculum plates were grown for 21 days and the percentage of agar in experimental plates was increased to 3%, an increase required to allow agar to solidify at pH 4.5 after the addition of gallic acid.

Reference

1. Justo, A., & Hibbett, D.S. Phylogenetic classification of *Trametes* (Basidiomycota, Polyporales) based on a five–marker dataset. *Taxon* **60**, 1567-1583 (2011)
